# Integrated 16S rRNA Sequencing, Metagenomics, and Metabolomics to Characterize Gut Microbial Composition, Function, and Fecal Metabolic Phenotype in Non-obese Type 2 Diabetic Goto-Kakizaki Rats

**DOI:** 10.3389/fmicb.2019.03141

**Published:** 2020-01-20

**Authors:** Weijun Peng, Jianhua Huang, Jingjing Yang, Zheyu Zhang, Rong Yu, Sharmeen Fayyaz, Shuihan Zhang, Yu-hui Qin

**Affiliations:** ^1^Department of Integrated Traditional Chinese and Western Medicine, The Second Xiangya Hospital, Central South University, Changsha, China; ^2^Hunan Academy of Chinese Medicine, Hunan University of Chinese Medicine, Changsha, China; ^3^ Hunan Key Laboratory of TCM Prescription and Syndromes Translational Medicine Hunan, Changsha, China; ^4^Department of Integrated Traditional Chinese and Western Medicine, Xiangya Hospital, Central South University, Changsha, China; ^5^Department of Gastroenterology, Xiangya Hospital, Central South University, Changsha, China; ^6^H.E.J. Research Institute of Chemistry, International Center for Chemical and Biological Sciences, University of Karachi, Karachi, Pakistan

**Keywords:** T2DM, GK rats, gut microbiome, fecal metabolomics, *Verrucomicrobia*, *Tenericutes*, glycerophospholipid metabolism

## Abstract

Type 2 diabetes mellitus (T2DM) is one of the most prevalent endocrine diseases in the world. Recent studies have shown that dysbiosis of the gut microbiota may be an important contributor to T2DM pathogenesis. However, the mechanisms underlying the roles of the gut microbiome and fecal metabolome in T2DM have not been characterized. Recently, the Goto-Kakizaki (GK) rat model of T2DM was developed to study the clinical symptoms and characteristics of human T2DM. To further characterize T2DM pathogenesis, we combined multi-omics techniques, including 16S rRNA gene sequencing, metagenomic sequencing, and metabolomics, to analyze gut microbial compositions and functions, and further characterize fecal metabolomic profiles in GK rats. Our results showed that gut microbial compositions were significantly altered in GK rats, as evidenced by reduced microbial diversity, altered microbial taxa distribution, and alterations in the interaction network of the gut microbiome. Functional analysis based on the cluster of orthologous groups (COG) and Kyoto Encyclopedia of Genes and Genomes (KEGG) annotations suggested that 5 functional COG categories belonged to the metabolism cluster and 33 KEGG pathways related to metabolic pathways were significantly enriched in GK rats. Metabolomics profiling identified 53 significantly differentially abundant metabolites in GK rats, including lipids and lipid-like molecules. These lipids were enriched in the glycerophospholipid metabolic pathway. Moreover, functional correlation analysis showed that some altered gut microbiota families, such as *Verrucomicrobiaceae* and *Bacteroidaceae*, significantly correlated with alterations in fecal metabolites. Collectively, the results suggested that an altered gut microbiota is associated with T2DM pathogenesis.

## Introduction

Type 2 diabetes mellitus (T2DM) is one of the most prevalent endocrine diseases and has become a major public health issue worldwide; it is expected to affect 693 million people worldwide by 2045 (Cho et al., 2018). T2DM is increasingly recognized as a multifactorial disorder influenced by genetic, environmental, and nutritional factors (Walker et al., 2014). Recent studies have indicated that gut microbial dysbiosis may partly induce T2DM development (Forslund et al., 2015; Yano et al., 2015). Qin et al. (2012) showed that patients with T2DM have a moderate degree of gut microbial dysbiosis. Wu et al. (2017) found that the composition and diversity of the gut microbiota in patients with T2DM exhibited significant changes after metformin treatment. These studies focused primarily on the composition and function of the fecal microbiome in individuals with diabetes. In recent years, research has demonstrated that microbiota-derived metabolites such as imidazole propionate, short-chain fatty acids (SCFAs), succinate, and *p*-cresol contribute to host insulin resistance (Koh et al., 2018; Canfora et al., 2019), which highlighted the associations between gut microbial metabolites and T2DM. However, no studies have comprehensively examined the compositional, functional, and metabolic dynamics of the diabetic microbiome. Thus, studies are needed to investigate the associations of the fecal microbiome with T2DM and further reveal the effects of fecal metabolic changes in disease pathogenesis.

The Goto-Kakizaki (GK) rat, a non-obese and spontaneous (genetic) T2DM experimental model, was generated from Wistar rats through repeated inbreeding of animals with impaired glucose tolerance resulting from impaired β-cell function on a background of polygenic inheritance (Zambrana et al., 2018). The advantages of GK rats include decreased β-cell numbers, impaired metabolic functions, reduced glucose-stimulated insulin secretion, glucose intolerance, and chronic inflammation (Guest, 2019; Ouyang et al., 2019). This model is frequently used to investigate the development of T2DM and its complications, since it could dissociate obesity related variables from the glucose homeostasis variable (Matsunaga et al., 2016; Sarkozy et al., 2016; Fu et al., 2019). The etiology of diabetes in GK rats was suggested to include genetic contributions and gestational metabolic impairment, resulting in epigenetic programing of offspring transmitted over generations, which causes reduced β-cell neogenesis and proliferation (Portha et al., 2010). These characteristics make GK rats an excellent experimental model.

The aim of current study was to systematically characterize global differences in fecal microbial communities, functions, and metabolic profiles of GK rats using 16S ribosomal RNA (16S rRNA) gene sequencing, metagenomics, and metabolomics, respectively. Our results clarify the pathogenesis and consequences of T2DM. This is the first report to evaluate gut microbiota composition and function and fecal metabolite profiles in GK rats.

## Materials and Methods

### Animal Model

Twenty 9-week-old male GK rats and 20 age-matched Wistar rats were obtained from Shanghai SLAC Laboratory Animal Co., Ltd. These rats were kept in the Laboratory of Animal Center, Hunan Academy of Chinese Medicine. Animals were individually housed under specific-pathogen free conditions at 23 ± 2°C, with a 12-h light-dark cycle under 50–60% atmospheric humidity and fed with regular rat chow and water *ad libitum*. The Animal Ethical Committee of Hunan Academy of Chinese Medicine approved all experimental procedures (approval no. 2018-0031). Body weight gain and fasting blood glucose (FBG) levels were measured weekly beginning at the age of 9 weeks.

### Fecal Sample Collection, DNA Extraction, and Metabolite Extraction

Fecal samples were collected at the age of 15 weeks, and at least 5 fecal pellets were obtained directly from the anus of each rat, deposited into a sterile conical tube, and immediately frozen at −80°C until further analysis. Microbial DNA was extracted as described in our previous study (Peng et al., 2018). DNA from fecal samples was isolated by using DNA E.Z.N.A.^®^ Stool DNA Kit (Omega Bio-Tek, Norcross, GA, United States) according to the manufacturer’s protocols. Total DNA quality was measured by using a spectrophotometer (NanoDrop 2000 UV; Thermo Fisher Scientific, Waltham, MA, United States) with 1% agarose gel electrophoresis.

Fecal metabolites were extracted following previously described procedures (Deda et al., 2018). Briefly, 50-mg fecal samples were accurately weighed, a volume of 400 μL ice-cold methanol/water (4:1, v/v) solution was added, and the mixture was allowed to settle at −20°C before homogenization using a mechanical disruptor (FastPrep-24^TM^5G, MP Biomedicals Co., Ltd., Shanghai, China) at 60 Hz for 6 min. The homogenate was vortexed for 15 min, sonicated for 10 min three times, then placed at −20°C for 30 min to precipitate proteins. After centrifugation at 13,000 × *g* at 4°C for 15 min, and filtration through a 0.22-μm membrane, the supernatant was prepared for LC–MS analysis.

### 16S rRNA Gene Sequencing Analysis

The 16S rRNA sequencing analysis approach was performed as described in our previous study (Peng et al., 2018). Briefly, PCR amplification was performed, purified amplicons were pooled, and paired-end sequenced was carried out. Then, the raw data was analyzed. The detailed sequencing analysis procedures are available in the Supplementary Material and Methods, and the data are deposited to the National Center for Biotechnology Information (NCBI) Sequence Read Archive (SRA) under BioProject number PRJNA588959.

### Metagenomic Analysis

Metagenomic sequencing of gut microbiota was conducted as described in our previous study (Peng et al., 2018). Briefly, microbial DNA was fragmented, metagenomic sequencing was performed, the clean raw reads were then assembled, open reading frames (ORFs) were predicted, and bioinformatic analysis was conducted. A more detailed analysis procedures is available in the Supplementary Material and Methods, and the data were deposited to the NCBI SAR under BioProject number PRJNA589664.

### Fecal Metabolic Analysis

Ultra-performance liquid chromatography coupled to triple quadrupole time-of-flight mass spectrometry (UPLC-Q-TOF-MS/MS) was used to analyze fecal metabolites as described in our previous study (Zhang et al., 2019b). Briefly, chromatographic separation was performed on Waters Acquity^TM^ UPLC system, mass spectrometry detection was triple TOF 5600 + MS/MS system (AB Sciex, Concord, ON, Canada). Mass data were collected in both positive and negative MSE continuum mode. Quality control (QC) samples were injected at regular intervals (every 10 samples). All raw data were imported into the Progenesis QI 2.3 (Nonlinear Dynamics, Waters, United States) and SIMCA-P + 14.0 software package for further data analysis. A more detailed analysis procedures is available in the Supplementary Material and Methods.

### Bioinformatics Analysis

All bioinformatics analyses were performed using the Majorbio Cloud Platform^1^. Diversity was calculated using the Quantitative Insights Into Microbial Ecology platform (QIIME) for 16S rRNA gene sequencing analysis results (Kuczynski et al., 2012). Chao, Simpsoneven, and Shannon indices were calculated to assess alpha diversity. Both the taxon-based Bray-Curtis distance and unweighted UniFrac phylogenetic distance were calculated to estimate beta diversity, and differences between two groups were visualized by principal coordinates analysis (PCoA) plots. Statistical significance was determined using analysis of similarities (ANOSIM). Statistically significant differences in the relative abundances of genera between GK and Wistar rats were determined using a linear discriminant analysis (LDA) effect size (LEfSe) algorithm (Segata et al., 2011). LDA values > 2.5 with a *P*-value < 0.05 were considered significantly enriched. NetworkX was used to explore and visualize associations between the microbial communities (Hagberg et al., 2008). The indexes of degree (DC), closeness (CC), and betweenness centrality (BC) were calculated to describe the topology features of constructed networks.

For metagenomic analysis, samples for sequencing were selected using microPITA (microbiomes: Picking Interesting Taxonomic Abundance) (Tickle et al., 2013). Significant differences in COG and KEGG categories between GK and Wistar rats were determined using LEfSe. Those with LDA values > 2.0 and *P* < 0.05 were considered significantly enriched.

For metabolomic analysis, principal component analysis (PCA) and orthogonal partial least squares discriminant analysis (OPLS-DA) algorithms were used to visually compare metabolite profiles. The importance of each metabolite was ranked according to their projection (VIP) scores calculated from the OPLS-DA model. Metabolic pathway analysis of these significantly different metabolites was performed using MetaboAnalyst software v.4.0 to identify the top altered pathways (Chong et al., 2018). Spearman correlation analysis was used to evaluate correlations between fecal metabolites and the gut microbiota.

## Results

### T2DM Characteristics of GK Rats

**FIGURE 1:**
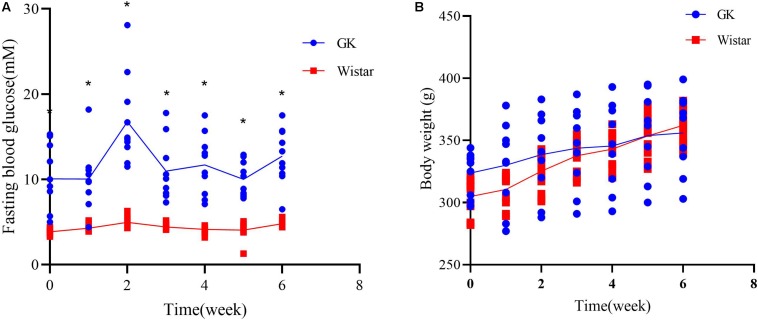
Diabetic characteristics of GK rats. **(A)** Fasting blood glucose levels were measured in GK and Wistar rats. **(B)** Body weight was assessed weekly in GK and Wistar rats. Data are expressed as the mean ± SEM. *n* = 10/group; ^∗^*p* < 0.0001.

To verify the development of T2DM in male GK rats, FBG levels were measured to assess glucose homeostasis each week. As expected, GK rats showed a significant increase in FBG levels at all time points compared to control Wistar rats (Figure 1A). There were no significant differences in body weight between two groups, confirming that the GK rats had a non-obese T2DM phenotype (Figure 1B). These findings demonstrate that GK rats presented with typical characterstics of T2DM.

### Alternative Gut Microbiota Composition in GK Rats

#### Structural Diversity of Gut Microbiota

**FIGURE 2:**
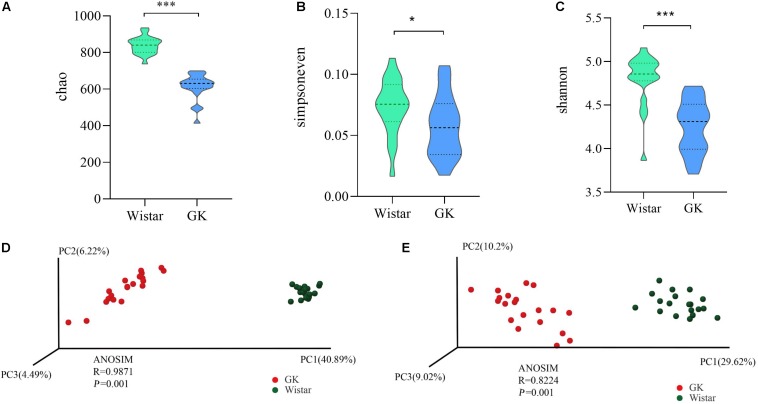
Gut microbial diversity in GK and Wistar rats. Alpha diversity was evaluated based on the Chao **(A)**, Simpsoneven **(B)**, and Shannon **(C)** indices of the OTU levels. ^∗^*P* < 0.05, ^∗∗∗^*P* < 0.001. Principal coordinates analysis of beta diversity was based on the weighted UniFrac **(D)** and Bray-Curtis **(E)** analyses of the OTU levels.

To investigate the variances of structural diversity of gut microbiota between GK and Wistar rats, we assessed microbial alpha diversity using the Chao, Simpsoneven, and Shannon indices to estimate richness, evenness, and diversity, respectively. We found that alpha diversity was significantly reduced in GK rats compared with Wistar rats, (*P* < 0.001 for the Chao richness index, Figure 2A; *P* = 0.046 for the Simpsoneven evenness index, Figure 2B; and *P* < 0.001 for the Shannon diversity index between two groups, Figure 2C). These results indicate that intra-individual bacterial diversity in GK rats distinctly differed from that in Wistar rats.

Moreover, the PCoA of weighted UniFrac distances and Bray–Curtis dissimilarity were used to measure beta diversity in each group. The results showed that the gut microbiota of GK rats was significantly different from that of Wistar rats in both weighted Uni-Frac distances (ANOSIM *R* = 0.9874, *P* = 0.001, Figure 2D) and Bray–Curtis dissimilarity (ANOSIM *R* = 0.8224, *P* = 0.001, Figure 2E). These results further indicated that beta diversity in GK rats was different from that of Wistar rats. That is, the structural diversity of the gut microbiota was significantly different in GK rats due to T2DM.

#### Altered Composition of the Gut Microbiota in GK Rats

**FIGURE 3:**
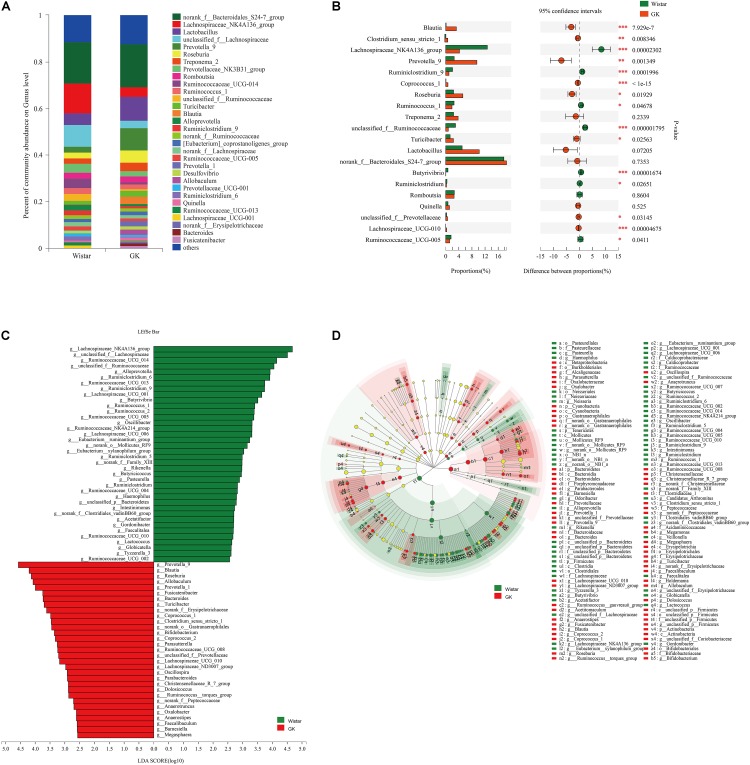
Gut microbiota composition profiles in GK and Wistar rats. **(A)** Summary of the relative abundances of bacterial genera detected in GK and Wistar rats. **(B)** Genus-level bacteria that were significantly different between the GK and Wistar rats. Data were showed as relative abundance (%) of top 20 most abundant genera in each group. Statistical analysis was performed by the Wilcoxon rank-sum test. ^∗∗∗^*P* < 0.001, ^∗∗^*P* < 0.01, and ^∗^*P* < 0.05 GK vs. Wistar group. **(C)** Cladogram generated from the LEfSe analysis indicating the phylogenetic distribution from phylum to genus of the microbiota of Wistar and GK rats. **(D)** Histogram of LDA scores to identify differentially abundant bacterial genera between GK and Wistar rats (LDA score ≥ 2.5).

As shown in Figure 3A, taxonomic analysis indicated that the relative abundance of 32 genera varied between GK and Wistar rats. Among these genera, *norank_f__ Bacteroidales_S24 -7_group* (18.55%) was the predominant genus in GK rats, followed by *Lactobacillus* (10.24%) and *Prevotella_9* (9.52%). In Wistar rats, the prevalent genera were *Norank_f__Bacteroidales_S247_group* (17.74%), *Lachnospiraceae_NK4A136_group* (12.70%), and *unclassified_ f__Lachnospiraceae* (9.25%).

Wilcoxon rank-sum tests were further performed to compare differences in fecal bacterial communities between the two groups at the genus level. The results revealed that 92 genera were significantly different between the two groups. Of these discriminatory taxa, *Prevotella_9*, *Lachnospiraceae_NK4A136_group*, *Roseburia*, *Blautia*, *unclassified_f__Lachnospiraceae*, *Turicibacter*, *Allobaculum*, and *Prevotella_1* were significantly more abundant in GK rats than in Wistar rats, whereas *Lachnospiraceae_NK4A136_group*, *Ruminococcaceae_UCG-014*, *unclassified_f__Lachnospiraceae*, and *unclassified_f__Ruminococcaceae* were significantly more abundant in Wistar rats (Figure 3B).

LEfSe was used to further determine whether specific bacterial taxa were differentially enriched in GK rats compared with Wistar rats. Using a logarithmic LDA score cutoff of 2.5, we identified 69 discriminatory genera as key discriminants (Figure 3C). Several genera including *Prevotella_9*, *Roseburia*, *Blautia, Turicibacter*, and *Allobaculum* were significantly overrepresented in the feces of GK rats, whereas *Lachnospiraceae_NK4A136_group*, *unclassified_f__Lachnospiraceae*, *Ruminococcaceae_UCG_014*, *unclassified_f__Ruminococcaceae*, and *Alloprevotella* were enriched in Wistar rats. A cladogram representing the taxonomic hierarchical structure of the fecal microbiota from phylum to species indicated significant differences in phylogenetic distributions between the microbiota of GK and Wistar rats (Figure 3D). These results showed a remarkable difference in fecal microbiota composition between GK and Wistar rats.

#### Correlation Network Analysis

**FIGURE 4:**
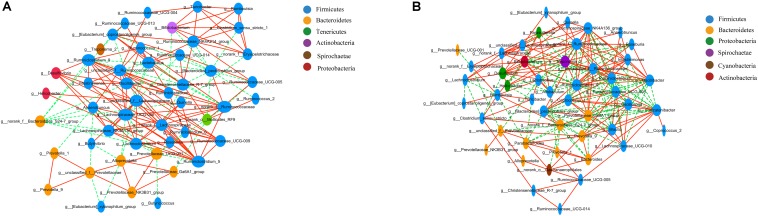
Correlation network analysis of the 50 most abundant genera for **(A)** Wistar and **(B)** GK rats. The lines between nodes indicate the Spearman correlation, and the color intensity indicates the correlation coefficient (red, positive; green, negative). Genera color are based on phylum affiliation, and sizes indicate mean relative abundance.

Correlation network analysis at the genus level was performed to determine whether T2DM was associated with changes in the correlation structure and putative interaction structure of the gut microbiota and to identify the putative keystone genera. We found that networks constructed from GK rat samples had more edges (225 vs. 166), a higher mean degree (9.33 vs. 6.87), and higher transitivity (0.585 vs. 0.439) that those constructed for Wistar rats. These results indicated that there were more significant correlations in GK rats than Wistar rats (Figures 4A,B). Moreover, DC, CC, and BC were determined to evaluate taxa importance within two networks. Based on the high scores of these topological properties (DC > 0.1, CC > 0.2, and BC > 0.1), one genus, *g__norank_f__Ruminococcaceae*, was identified in GK rats. Four genera were identified in Wistar rats: *Lachnospiraceae_UCG-006*, *norank_f__Ruminococcaceae*, *Prevotellaceae_UCG-001*, and *unclassified_f__Lachnospiraceae*.

### Metagenomic Analysis Revealed Different Functional Profiles

#### microPITA Analysis

**FIGURE 5:**
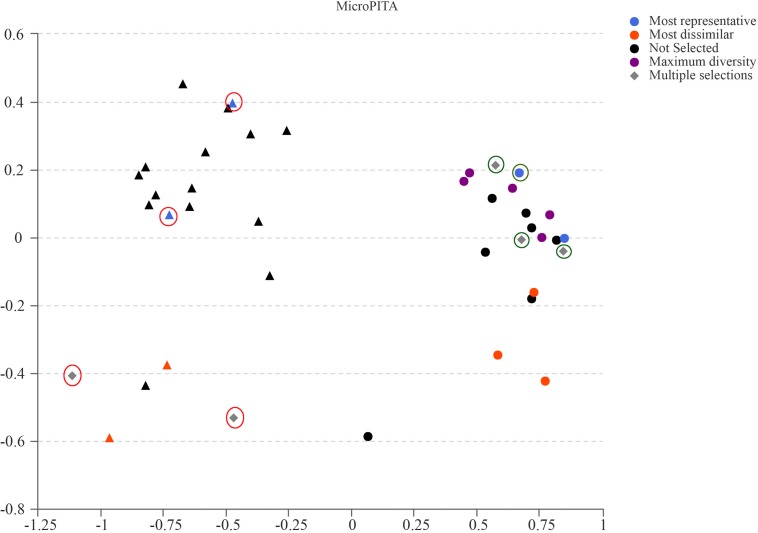
MicroPITA (microbiomes: Picking Interesting Taxonomic Abundance) analysis. Principle coordinates analysis using Bray–Curtis dissimilarity for eight samples (four for GK rats and four for Wistar) selected by each of the four unsupervised criteria. Red and green circles represent the selected samples of GK and Wistar rats, respectively.

Samples for metagenomic sequencing were selected from the 40 samples of 16S data using the microPITA method (Tickle et al., 2013). Based on the term “most dissimilar (samples with the most extreme microbial communities in the survey),” “most representative (samples with microbial communities representative of the survey),” “maximum diversity (samples with the most diverse community),” and “multiple selections (samples with two or more above-mentioned characters),” four GK fecal samples and four Wistar fecal samples were selected for further investigation (Figure 5).

#### COG Annotation and Analysis

**FIGURE 6:**
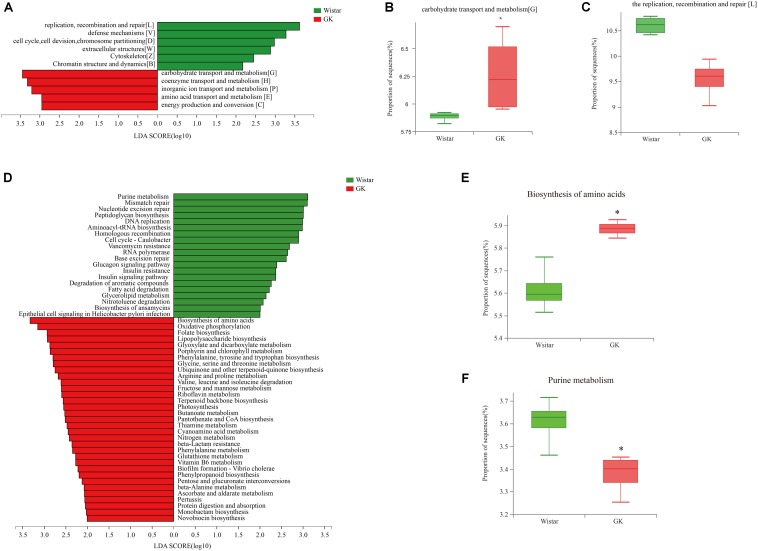
Linear discriminant analysis (LDA) integrated with effect size (LEfSe) analysis. **(A)** LDA integrated with LEfSe comparison of relative abundances of cluster of orthologous groups (COG) categories between GK and Wistar rats (LDA score > 2.0, *P* < 0.05). **(B,C)** Differences in relative abundances of COG categories **[L]** and **[G]**. **(D)** LDA integrated with LEfSe comparison of relative abundance of KEGG pathways between GK and Wistar rats (LDA score > 2.0, *P* < 0.05). **(E)** The relative abundance of the purine metabolic pathway was significantly enriched in Wistar rats. **(F)** The biosynthesis of amino acids pathway was significantly enriched in GK rats.

LEfSe analysis was performed to identify biologically significant differences in functional COG categories between GK and Wistar rats. This analysis provided insights into the functional properties of fecal microbes. As shown in Figure 6A, we found 11 significantly different functional COGs between GK and Wistar rats. Of these COG categories, five functional COG categories were highly enriched in the GK group, including coenzyme transport and metabolism [H], energy production and conversion [C], amino acid transport and metabolism [E], carbohydrate transport and metabolism [G] (Figure 6B), and inorganic ion transport and metabolism [P]. In contrast, the metagenome of Wistar rats were enriched in replication, recombination, and repair [L] (Figure 6C); defense mechanisms [V]; cell cycle control, cell division, chromosome partitioning [D]; extracellular structures [W]; cytoskeleton [Z]; and chromatin structure and dynamics [B]. Notably, the predominant COG categories associated with GK rats were in the metabolism cluster.

#### Kyoto Encylopedia of Genes and Genomes Functional Annotation and Analysis

Linear discriminant analysis effect size analysis was performed to explore KEGG pathways with significantly different abundances between GK and Wistar rats (Figure 6D). Based on the threshold values LDA > 2.5 and *P* < 0.05, 27 KEGG pathways (including biosynthesis of amino acids (Figure 6E), oxidative phosphorylation, folate biosynthesis, lipopolysaccharide biosynthesis, and others) were significantly enriched in GK rats, and 11 KEGG pathways (including purine metabolism (Figure 6F), mismatch repair, nucleotide excision repair, peptidoglycan biosynthesis, DNA replication, and others) were significantly increased in Wistar rats. Multiple functional pathways that were more highly represented in GK rats than in Wistar rats were involved in metabolism.

### Alterations in the Fecal Metabolic Profile of GK Rats

#### Multivariate Statistical Analysis

**FIGURE 7:**
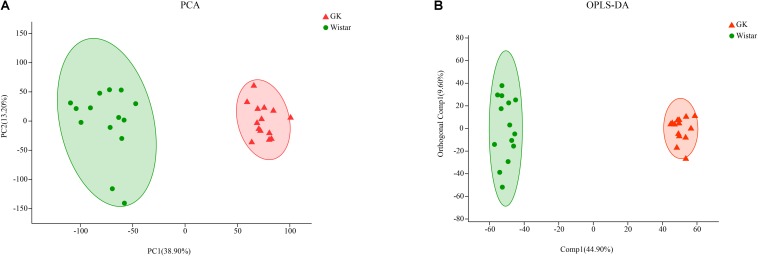
Multivariate statistical analysis of fecal metabolites in GK and Wistar rats. **(A)** PCA and **(B)** OPLS-DA plots showing spatial division between GK and Wistar rats.

Principal component analysis (PCA) algorithm was used distinguish the inherent trends within the metabolic data of GK rats and Wistar rats. As shown in Figure 7A, differences were observed between the two groups, which indicated inherent metabolic differences between them.

To further identify metabolites that discriminate between GK rats and Wistar rats, an orthogonal partial least squares-discriminant analysis (OPLS-DA) model was constructed. The OPLS-DA score plot showed clear discrimination between two groups with [*R*^2^X (cum) = 0.435, *R*^2^Y (cum) = 0.977, *Q*^2^(cum) = 0.972], which suggested that the model was predictive and reliable, and that differences in metabolites’ abundance between GK and Wistar rats were highly significant (Figure 7B).

### Metabolic Variation Analysis in GK Rats

**FIGURE 8:**
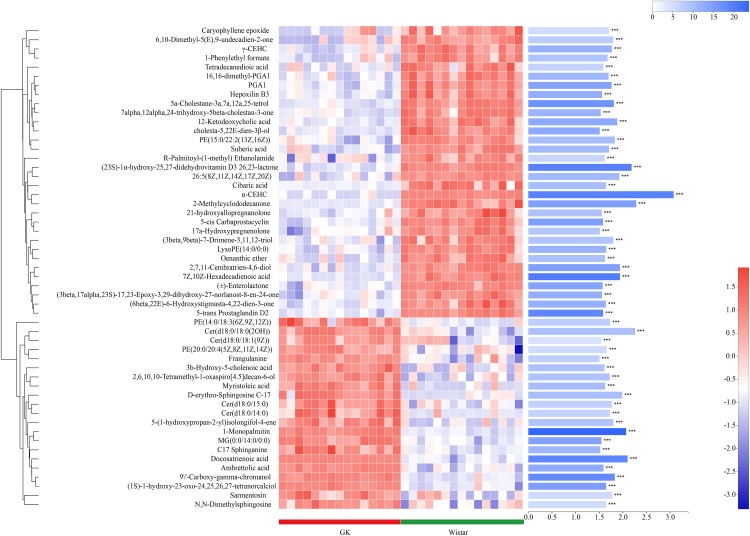
Fecal metabolic profiles in GK and Wistar rats. Hierarchical clustering and heat map in the left panel showing the 53 metabolites that were significantly differentially abundant between GK and Wistar rats. Each row represents data for a specific metabolite, and each column represents an individual. Different colors correspond to different metabolite abundance levels. Red and blue colors represent increased and decreased levels of metabolites, respectively. The histogram in the right panel represents variable importance in projection (VIP) scores derived from the OPLS-DA model for each metabolite. ^∗∗∗^ Indicates *P* < 0.001.

Metabolites with VIP scores > 1 in the multivariate model OPLS-DA and *p* < 0.05 were considered as potential metabolic biomarkers (Supplementary Table S1). Metabolites with VIP > 1.5 in the multivariate model OPLS-DA and *p* < 0.05 in the univariate analysis were selected as significantly differentially abundant metabolites (Supplementary Table S2). A total of 169 potential metabolic biomarkers and 53 significantly differentially abundant metabolites were identified between GK and Wistar rats. A heat map was constructed to visualize these 53 significantly differentially abundant metabolites (Figure 8). Overall, 22 and 33 metabolites were significantly higher and lower in GK rats, respectively. Most of the differentially abundant metabolites were lipids and lipid-like molecules. Several sterol lipids such as 7α,12α,24-trihydroxy-5β-cholestan-3-one, 21-hydroxyallopregnanolone, and (23S)-1α-hydroxy-25,27-didehydrovitamin D3 26,23-lactone were among the metabolites downregulated in GK rats. Others glycerophospholipids such as PE[20:0/20:4 (5Z,8Z,11Z,14Z)] and PE [14:0/18:3(6Z,9Z,12Z)] were present at higher levels in GK rats.

### Correlation Analysis of Gut Microbiota and Fecal Metabolic Phenotype

**FIGURE 9:**
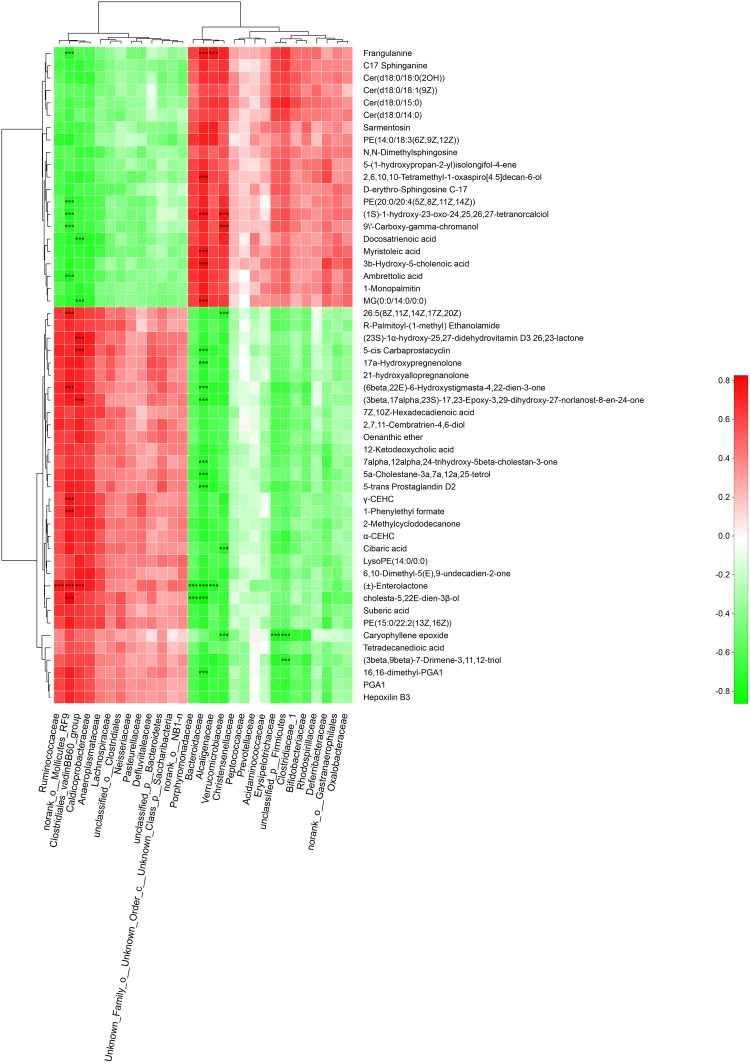
Spearman’s correlation analysis between the gut bacteria phyla and altered fecal metabolites. Positive and negative correlations are shown as red and green in the heat map, respectively. Significant microbiota-metabolite correlations were determined based on an | *r* | ≥ 0.75 and *P* < 0.01 (^∗∗∗^*P* < 0.001).

To explore the functional correlation between gut microbiota dysbiosis and altered fecal metabolites, a correlation matrix was calculating by using the Spearman’s correlation coefficients between microbial communities at the family level (29 bacterial taxa) and the 53 significantly altered metabolites (VIP > 1.5). As shown in Figure 9 a total of 46 significant microbiota-metabolite correlations were determined based on an |*r*| ≥ 0.75 and *P* < 0.01. Specifically, *norank_o__ Mollicutes_RF9*, *lostridiales_vadinBB60_group*, *Bacteroidaceae* and *Verrucomicrobiaceae* were significantly associated with 11, 6, 16, and 6 fecal metabolites, respectively. In addition, *unclassified_p__Firmicutes* was negatively correlated with caryophyllene epoxide and (3beta,9beta)-7-Drimene-3,11,12-triol. *Erysipelotrichaceae* was negatively correlated with caryophyllene epoxide. *Ruminococcaceae* and *Alcaligenaceae* were positively and negatively correlated with (±)-Enterolactone, respectively. These correlation data suggested GK rats exhibited significant taxonomic perturbations in the gut microbiome, which may result in a significantly altered metabolomic profile.

## Discussion

This was the first study to characterize the fecal microbiome of GK rats by integrating 16S rRNA gene sequencing, metagenomic sequencing, and LC–MS-based metabolomics approaches. Our results showed that the gut microbiota composition and function and fecal metabolic phenotype were significantly different in GK rats compared to Wistar rats.

Reduced alpha and beta diversity and altered gut microbiota composition were observed in GK rats compared to Wistar rats based on 16S rRNA gene sequencing results. This suggested that T2DM may be linked to dynamic changes of the compositions of intestinal microbiota (Zhou et al., 2019). Compared to Wistar rats, GK rats exhibited significantly lower proportions of the phyla Firmicutes, Saccharibacteria, and Tenericutes, and significantly higher proportions of Bacteroidetes, Deferribacteres, and Actinobacteria. Moreover, the phylum Proteobacteria was also significantly increased, in GK rats compared to Wistar rats. These results were consistent with a previous study (Larsen et al., 2010). We also found that the proportion of the phylum Firmicutes was decreased, and the proportions of the phyla Bacteroidetes and Proteobacteria were increased, in rats with T2DM than those in non-diabetic controls. Firmicutes, the most abundant bacterial phylum of the gut microbiota in GK rats, could potentially have effects on the production of short-chain fatty acids (SCFAs) (Duncan et al., 2007). SCFAs exert significant physiological and pharmacological effects, and they are regarded as nutritional targets to prevent or treat T2DM (Hu et al., 2018). Bacteroidetes and Proteobacteria, the gram-negative bacteria, could produce lipopolysaccharides (LPS), and subsequently trigger an inflammatory response and contribute to the development of diabetes (Larsen et al., 2010). The phylum Deferribacteres is involved in the iron metabolism (Li et al., 2019), and abnormal iron metabolism are associated with a greater risk of type 2 diabetes mellitus (Fernandez-Real et al., 2015). In addition, correlation network analysis indicated perturbation of the gut microbiota interaction network in GK rats. A similar disruption of the gut microbiota interaction network was also observed in Alzheimer’s disease transgenic mice (Peng et al., 2018) and patients with gastric cancer (Chen et al., 2019b). Further studies are needed to characterize the role of the gut microbiota interaction network in T2DM progression.

Metagenomic sequencing has been widely employed for comprehensive analysis of the relationships between microbial function and host physiology. Several studies have employed metagenomic approaches to explore novel changes in the functional potential of the microbiota (Qin et al., 2012; Wei et al., 2013; Lee et al., 2018). The results of COG and KEGG functional analysis showed that disruptions in gut microbiota function were mainly associated with perturbed metabolic pathways. We found that five functional COG categories were enriched in GK rats, including energy production and conversion, coenzyme, amino acid, carbohydrate, and inorganic ion transport and metabolism. KEGG analysis further indicated that these perturbed gut bacteria in GK rats were strongly associated with the dysregulation of some metabolic processes such as glyoxylate and dicarboxylate metabolism; porphyrin and chlorophyll metabolism; and glycine, serine, and threonine metabolism. Interestingly, the five COG categories enriched in our study, in addition to glyoxylate and dicarboxylate metabolism, have been implicated in the antidiabetic effects of metformin (Dong et al., 2016; Chen et al., 2018).

Fecal metabolome characterization can improve understanding of microbial responses to gut microbiota perturbations. The fecal metabolic profiles were significantly different between GK and Wistar rats. A total of 53 fecal metabolites were identified as biomarkers of T2DM with VIP > 1.5 in OPLS-DA and *p* < 0.05. Furthermore, five perturbed metabolic pathways were identified in GK rats (Supplementary Figure S1). Although there are only three metabolites matched to glycerophospholipid metabolic pathway, we presume that this pathway may perturbed in GK rats (Supplementary Table S3). This metabolic pathway was also shown to be the main disordered pathway in serum samples from patients with T2DM (Zhao et al., 2017). As the major components of cell membranes, glycerophospholipids have been closely associated with insulin resistance and T2DM (Pantophlet et al., 2017). Disturbances in membrane glycerophospholipid metabolism would influence insulin secretion, further affecting the metabolic of carbohydrates and lipids (Nolan et al., 2006). Moreover, the glycerophospholipid metabolic pathway could be used as a therapeutic target of T2DM-induced dementia in db/db mice (Niu et al., 2015), high-fat diet-induced T2DM in C57BL/6 mice (Chen et al., 2019a), and T2DM in humans (Liu et al., 2018). Of course, the related metabolites of glycerophospholipid metabolism pathway need to quantitatively analysis by the targeted metabolomics, and the role of this pathway in T2DM pathogenesis should also be determine in the further studies.

A significant correlation between gut microbiota families and fecal metabolites was observed, which indicated that gut microbiota perturbations were associated with metabolic phenotype alterations. Of particular interest, we found that the families *Verrucomicrobiaceae* and *Bacteroidaceae* were also dysregulated in the diabetes mouse model (Zhang et al., 2019a). The family *Verrucomicrobiaceae* belongs to the phylum Verrucomicrobia, significantly increased and has been shown to be associated with elevated plasma concentrations of tumor necrosis factor α (TNF-α) and interferon γ in patients with Parkinson’s disease (Lin et al., 2019). The family *Bacteroidaceae*, belongs to the phylum Bacteroidetes, was found to be decreased with aging and were inversely correlated with colonic proinflammatory cytokines, including TNF-α, interleukin-1β, and interleukin-6 (Kim et al., 2019). These findings implied that the families *Verrucomicrobiaceae* and *Bacteroidaceae* and their associated fecal metabolites may contribute to inflammation associated with T2DM.

Our results should be considered in the context of several limitations. First, the animal and metagenomic sample sizes were small, and larger cohorts should be assessed in future studies. Second, we did not characterize associations between host functions and the microbiome. Host metabolomics data or physiological parameters are essential for characterizing host–microbiota interactions. Third, absolute, rather than relative, quantification of microbial abundances might be a better indicator of T2DM pathogenesis (Vandeputte et al., 2017). Finally, the roles of the most relevant taxa remain to be investigated.

## Conclusion

We observed dynamic shifts in the compositions and functions of gut microbes and fecal metabolites in GK rats. Multiple metabolic pathways were significantly associated with T2DM. In particular, the glyoxylate, dicarboxylate, and glycerophospholipid metabolic pathways may serve as potential therapeutic targets for T2DM. Some altered gut microbiota phyla such as Verrucomicrobia and Tenericutes were strongly correlated with alterations in fecal metabolite abundance. Our results demonstrate concurrent changes in the microbiota and functional capacity during the progression of T2DM in the GK rat model. Future studies should assess the longitudinal microbiota before onset as well as during the development of T2DM and broaden the analysis to evaluate host response and metatranscriptomics to get more complete picture of the pathogenesis of T2DM, with the hope of identifying targets for drug development.

## Data Availability Statement

The data collected in the present study were properly analyzed and summarized in the Results section. The raw data were deposited in NCBI Sequence Read Archive (SRA) (accession numbers for NCBI: BioProject: PRJNA589664 for Metagenomic data; BioProject: PRJNA588959 for 16S rRNA sequencing).

## Ethics Statement

The animal study was reviewed and approved by The Animal Ethical Committee of Hunan Academy of Chinese Medicine.

## Author Contributions

WP conceived and designed the work. WP and JH coordinated technical support and funding. WP, JH, and SF wrote the manuscript. JY, ZZ, and SZ performed the experiments and collected the samples. WP and JH acquired, analyzed, and interpreted the data. RY, Y-HQ, and SZ reviewed the manuscript. All authors read and approved the final manuscript.

## Conflict of Interest

The authors declare that the research was conducted in the absence of any commercial or financial relationships that could be construed as a potential conflict of interest.

## Footnotes

1 www.majorbio.com

## Supplementary Material

FIGURE S1 Perturbed Metabolic Pathways in GK rats.

TABLE S1 The different fecal metabolites with VIP > 1 and *P* < 0.05 between GK rats and Wistar rats.

TABLE S2 The different fecal metabolites with VIP > 1.5 and *P* < 0.05 between GK rats and Wistar rats.

TABLE S3 MetaboAnalyst V.4.0 Pathway analysis in fecal samples of GK and Wistar rats.
