# Supplementary material for: Integrated 16S rRNA Sequencing, Metagenomics, and Metabolomics to Characterize Gut Microbial Composition, Function, and Fecal Metabolic Phenotype in Non-obese Type 2 Diabetic Goto-Kakizaki Rats
Source: Front Microbiol. 2020 Jan 20;10:3141. doi: 10.3389/fmicb.2019.03141 (PMC6984327; doi:10.3389/fmicb.2019.03141)
Supplement: FIGURE S1 — Perturbed Metabolic Pathways in GK rats. [file Data_Sheet_1.DOCX]

**Supplementary Material and Methods**

**1 16S rRNA gene sequencing analysis**

16S rRNA sequencing analysis approach was performed as described in our previous study (Peng et al., 2018). Firstly, PCR was performed to amplification of the V3-V4 hypervariable regions of the bacterial 16S rRNA gene according to universal primers (338F 5′-ACTCCTACGGGAGGCAGCAG-3′, 806R 5′-GGACTACHVGGGTWTCTAAT-3′) that contained FLX Titanium adaptors and a barcode sequence. Then, purified amplicons were pooled in equimolar amounts, and paired-end sequenced by using an Illumina MiSeq platform (Illumina, San Diego, USA). Raw FastQ files were demultiplexed, quality filtered using Trimmomatic, and merged using FLASH. Trimmed sequences were clustered to operational taxonomic units (OTUs) with a 97% similarity cut-off using UPARSE (version 7.1 http://drive5.com/uparse/), and chimeric sequences were identified and removed using UCHIME. Taxonomical assignment of OTUs was performed using the RDP Classifier algorithm (http://rdp.cme.msu.edu/) against the Silva database (https://www.arb-silva.de/) with a confidence threshold set as 70%. The sequence data are deposited to the National Center for Biotechnology Information (NCBI) Sequence Read Archive (SRA) under BioProject number PRJNA588959.

**2 Metagenomic analysis**

Metagenomic sequencing of gut microbiota was conducted as described in our previous study (Peng et al., 2018). Firstly, microbial DNA was fragmented to an average size of approximately 300 bp using TruSeq™ DNA Sample Prep Kit with Covaris M220 (Gene Company Limited, China) for paired-end library construction. Metagenomic sequencing was performed using an Illumina HiSeq4000 sequencing platform (Illumina Inc., San Diego, CA, USA). Raw sequence reads with a quality score lower than 20 and a length shorter than 50 bases were discarded. The clean raw reads were then assembled using SOAPdenovo software to obtain contigs for prediction and annotation. Open reading frames (ORFs) from each sample were predicted using MetaGene (http://metagene.nig.ac.jp/). The cluster of orthologous groups of proteins (COG) annotation of the ORFs was obtained using the eggNOG database (Version 4.5) via BLASTP (BLAST Version 2.2.28+) with an e-value cutoff of 1e-5. KEGG pathway annotation was performed by using a BLAST search (Version 2.2.28+) against the KEGG database (http://www.genome.jp) at an optimized e-value cutoff of 1e-5. The metagenomic sequence data are **deposited** to the NCBI SAR under BioProject number PRJNA588959.

**3 Fecal metabolic analysis**

Ultra-performance liquid chromatography coupled to triple quadrupole time-of-flight mass spectrometry (UPLC-Q-TOF-MS/MS) was used to analyze fecal metabolites as described in our previous study(Zhang et al., 2019). Briefly, chromatographic separation was performed on Waters Acquity™ UPLC system equipped with a BEH C18 column (100 mm × 2.1 mm i.d., 1.7 µm). The mobile phases were 0.1% formic in water (solvent A), and acetonitrile/isopropanol (v/v, 1:1) with 0.1% formic acid (solvent B). The flow rate was 0.400 mL/min. Mass spectrometry detection was triple TOF 5600+ MS/MS system (AB Sciex, Concord, Ontario, Canada). The ion source temperature was 110 °C, and the desolvation temperature was 450 °C, positive- and negative-ion electrospray ionization (ESI) modes were detected. The capillary voltage, and sample cone voltage were set to 2.5 kV, and 40 V, respectively. Nitrogen was used as both the desolvation gas (900 L/h) and the cone gas (50 L/h). Mass data were collected from the range of m/z 50-1500 in both positive and negative MSE continuum mode. Quality control (QC) samples were firstly prepared by mixing all the samples from each group, and injected at regular intervals (every 10 samples) throughout the whole analytical procedure to validate data quality.

All raw data were imported into the Progenesis QI 2.3 (Nonlinear Dynamics, Waters, USA) for peak detection and alignment. The preprocessing results generated a data matrix that consisted of the retention time (RT), mass-to-charge ratio (m/z) values, and peak intensity. Metabolic features detected at least 50 % in any set of samples were retained. After filtering, minimum metabolite values were imputed for specific samples in which the metabolite levels fell below the lower limit of quantitation and each metabolic feature were normalized by sum. The internal standard was used for data QC (reproducibility), metabolic features which the relative standard deviation (RSD) of QC>30% were discarded. Following normalization procedures and imputation, statistical analysis was performed on log transformed data to identify significant differences in metabolite levels between comparable groups. Mass spectra of these metabolic features were identified by using the accurate mass, MS/MS fragments spectra and isotope ratio difference with searching in reliable biochemical databases as Human metabolome database (HMDB) (http://www.hmdb.ca/) and Metlin database (https://metlin.scripps.edu/). Concretely, the mass tolerance between the measured m/z values and the exact mass of the components of interest was ±10ppm. For metabolites having MS/MS confirmation, only the ones with MS/MS fragments score above 30 were considered as confidently identified. Otherwise, metabolites had only tentative assignments. Metabolite identification levels were assigned based on the Metabolomics Standards Initiative criteria as follows(Sumner et al., 2007): (1) confidently identified compounds (level 1); putatively annotated compounds (level 2). Finally, positive and negative mode data were combined, and inputted to the SIMCA-P+ 14.0 software package for data analysis (Umetrics, Umeå, Sweden).

**References**

Peng, W., Yi, P., Yang, J., Xu, P., Wang, Y., Zhang, Z., et al. (2018). Association of gut microbiota composition and function with a senescence-accelerated mouse model of Alzheimer's Disease using 16S rRNA gene and metagenomic sequencing analysis. *Aging (Albany NY)* 10(12)**,** 4054-4065. doi: 10.18632/aging.101693.

Sumner, L.W., Amberg, A., Barrett, D., Beale, M.H., Beger, R., Daykin, C.A., et al. (2007). Proposed minimum reporting standards for chemical analysis Chemical Analysis Working Group (CAWG) Metabolomics Standards Initiative (MSI). *Metabolomics* 3(3)**,** 211-221. doi: 10.1007/s11306-007-0082-2.

Zhang, Z., Yi, P., Yang, J., Huang, J., Xu, P., Hu, M., et al. (2019). Integrated network pharmacology analysis and serum metabolomics to reveal the cognitive improvement effect of Bushen Tiansui Formula on Alzheimer's disease. *Journal of Ethnopharmacology***,** 112371. doi: <https://doi.org/10.1016/j.jep.2019.112371>.





**Figure S1.** **Perturbed Metabolic Pathways in GK rats.**

To identify metabolic pathways that were perturbed in GK rats, we performed metabolic pathway analysis of the 169 potential metabolic biomarkers using MetaboAnalyst v.4.0 software. The perturbed metabolic pathways in the fecal samples are shown in Supplementary Table S3, the results indicated that five metabolic pathways including phenylalanine, tyrosine and tryptophan biosynthesis; glycerophospholipid metabolism; sphingolipid metabolism; tyrosine metabolism; and steroid hormone biosynthesis were perturbed in GK rats, with impact values > 0.01. The glycerophospholipid metabolism pathway was considered the most relevant pathway in GK rats based on an impact value > 0.1 and p  < 0.05. All matched pathways are displayed as circles. The size and color of each circle is based on the pathway impact value and P-value, respectively. Perturbed pathways with impact values > 0.01 are annotated.
