# Supplementary material for: Integrated 16S rRNA Sequencing, Metagenomics, and Metabolomics to Characterize Gut Microbial Composition, Function, and Fecal Metabolic Phenotype in Non-obese Type 2 Diabetic Goto-Kakizaki Rats
Source: Front Microbiol. 2020 Jan 20;10:3141. doi: 10.3389/fmicb.2019.03141 (PMC6984327; doi:10.3389/fmicb.2019.03141)
Supplement: TABLE S3 — MetaboAnalyst V.4.0 Pathway analysis in fecal samples of GK and Wistar rats. [file Table_3.DOCX]

**Table S3. MetaboAnalyst V.4.0 Pathway analysis in fecal samples of GK and Wistar rats**

| Pathway Name | Match Status | p | -log(p) | Holm p | FDR | Impact |
| --- | --- | --- | --- | --- | --- | --- |
| Phenylalanine, tyrosine and tryptophan biosynthesis | 1/4 | 0.079753 | 2.5288 | 1 | 1 | 0.5 |
| Glycerophospholipid metabolism | 3/36 | 0.035451 | 3.3396 | 1 | 1 | 0.21631 |
| Sphingolipid metabolism | 1/21 | 0.35522 | 1.035 | 1 | 1 | 0.15416 |
| Tyrosine metabolism | 1/42 | 0.58687 | 0.53294 | 1 | 1 | 0.13972 |
| Steroid hormone biosynthesis | 2/77 | 0.47598 | 0.74238 | 1 | 1 | 0.05875 |
| Glycosylphosphatidylinositol (GPI)-anchor biosynthesis | 1/14 | 0.25314 | 1.3738 | 1 | 1 | 0.00399 |
| Linoleic acid metabolism | 1/5 | 0.098708 | 2.3156 | 1 | 1 | 0 |
| Ubiquinone and other terpenoid-quinone biosynthesis | 1/9 | 0.17082 | 1.7671 | 1 | 1 | 0 |
| Phenylalanine metabolism | 1/12 | 0.22121 | 1.5087 | 1 | 1 | 0 |
| alpha-Linolenic acid metabolism | 1/13 | 0.23733 | 1.4383 | 1 | 1 | 0 |
| Lysine degradation | 1/25 | 0.40735 | 0.89808 | 1 | 1 | 0 |
| Arachidonic acid metabolism | 1/36 | 0.53054 | 0.63387 | 1 | 1 | 0 |
| Tryptophan metabolism | 1/41 | 0.57796 | 0.54825 | 1 | 1 | 0 |
| Primary bile acid biosynthesis | 1/46 | 0.62074 | 0.47685 | 1 | 1 | 0 |
| Aminoacyl-tRNA biosynthesis | 1/48 | 0.63664 | 0.45154 | 1 | 1 | 0 |
